# Characterization and phylogenetic analyses of the complete mitochondrial genome of *Sillaginopsis panijus* (Perciformes: Sillaginidae)

**DOI:** 10.1080/23802359.2021.1989339

**Published:** 2021-10-14

**Authors:** Shilpi Saha, Na Song, Mohammad Abdul Baki, Tianyan Yang, Tianxiang Gao

**Affiliations:** a Fisheries College, Ocean University of China, Shandong, China; b Department of Zoology, Jagannath University, Dhaka, Bangladesh; c Fishery College, Zhejiang Ocean University, Zhoushan, China

**Keywords:** Bay of Bengal, mitogenome, Sillaginidae, *Sillaginopsis panijus*

## Abstract

The complete mitochondrial genome of *Sillaginopsis panijus* has been determined for the first time using Sanger Dideoxy DNA sequencing. The mitogenome is a circular molecule of 16,529 bp in length. It contains 37 mitochondrial genes (13 protein-coding genes, two ribosomal RNA, and 22 transfer RNA) and a control region as other bony fishes. In the phylogenetic analysis using 12H-strand protein-coding genes, monotypic *S*. *panijus* is situated separately from the genus *Sillago*. The present phylogeny supports its taxonomic position according to morphology and will be helpful for evolutionary analysis.

The Gangetic whiting, *Sillaginopsis panijus* (Hamilton-Buchanan 1822), is the only species of the genus *Sillaginopsis*. It is well documented as a commercial fish species due to its deliciousness, white flesh and few bones. It is distributed in the Northeastern Indian Ocean, including India, Bangladesh and Myanmar (Bay of Bengal), southward to Malaysia, and hardly to the Indonesian Archipelago (Andaman Sea) (McKay 1992). Its taxonomic description (Dutt and Sujatha 1980) and phylogeny (Kaga 2013) are mainly based on morphological characteristics. However, there were only partial COI and 12S rRNA sequences available in GenBank. Later an incomplete mitogenome (accession no. AP006802) (without a control region) was made available in GenBank before the release of data of this study. The present study sequenced and characterized its mitogenome and explored the phylogenetic relationship. The study will give supportive genetic information for future research in genetics and phylogenetics.

The specimen was collected from the Cox's Bazar, Bangladesh (21.452° N, 91.964° E) on 2nd October 2018. Muscle tissue (Voucher number, FEL_OUC142276) was deposited in the Fishery Ecology Laboratory, Fisheries College, Ocean University of China (http://eweb.ouc.edu.cn, Jia guang Xiao is the contact person 1099869663@qq.com). Genomic DNA was extracted by proteinase K digestion followed by a standard phenol-chloroform method (Sambrook et al. 1989). A PCR-based primer walking method was used to ascertain the mitogenome. SeqMan (DNAStar, USA) software was used to manually correct and align the sequences. The start and end positions of each gene were determined by comparing with the complete mitochondrial DNA sequences of the Sillaginidae species published in GenBank. The tRNAscan-SE 2.0 (Lowe and Chan 2016) was used to identify tRNA genes. The location of the control area was determined through the position of *tRNA-Phe* and *tRNA-Pro*.

Maximum likelihood (ML) and Bayesian inference (BI) methods were carried out on concatenated sequences of protein-coding genes (PCGs) (except *ND6*) of eight Sillaginidae species to reveal the phylogenetic relationship. ClustalX 2.1 (Larkin et al. 2007) was used to align the sequences. By comparing the Akaike Information Criterion value in jModelTest v2.1.10 (Darriba et al. 2012), the GTR + I + G model was selected as the best to describe the substitution pattern. The PAUP* 4.0 (Swofford 2002) was used to perform ML analysis with 1000 bootstrap replications. Three partitioned (first, second and third codon positions of PCGs) BI analysis was done by MrBayes v. 3.2.6 (Ronquist et al. 2012).

The mitogenome of *S. panijus* was sequenced to be 16,529 bp in length (GenBank accession no. MT460675). It consists of 13 typical vertebrate PCGs, 22 transfer RNA genes, two ribosomal RNA genes (12S rRNA and 16S rRNA), and two non-coding regions (control region and L-strand replication origin). It has shown the similarity with the canonical organization of the fish mitogenome in both gene content and order (Boore 1999). The encoding genes were located on the heavy strand with the exception of *ND6* and eight tRNA (*tRNA-Gln*, *tRNA-Ala*, *tRNA-Asn*, *tRNA-Cys*, *tRNA-Tyr*, *tRNA-Ser*, *tRNA-Glu* and *tRNA-Pro*) genes were transcribed from the light strand. The overall nucleotide composition was estimated to be 27.97% T, 27.85% C, 26.18% A and 18.00% G, with anti-G bias and a slight excess of AT (54.15%). The 13 PCGs were a total of 11,443 bp in size, accounting for 69.23% of the whole mitogenome. All PCGs except *COI* use ATG as the start codon, while *COI* uses GTG as the start codon. Seven PCGs have the complete stop codon TAA (*COI*, *ATPase8*, *COIII*, *ND4L* and *ND6*) or TAG (*ND1* and *ND5*). While the other six have the incomplete stop codon TA (*ND2*, *ATPase6*) or T (*COII*, *ND3*, *ND4* and *Cytb*). In the phylogenetic tree, species of the genus *Sillago* clustered together. On the contrary, *S. panijus* was situated outside the clade of the genus *Sillago* that supported its morphological division at the genus level (Figure 1).

**Figure 1.**
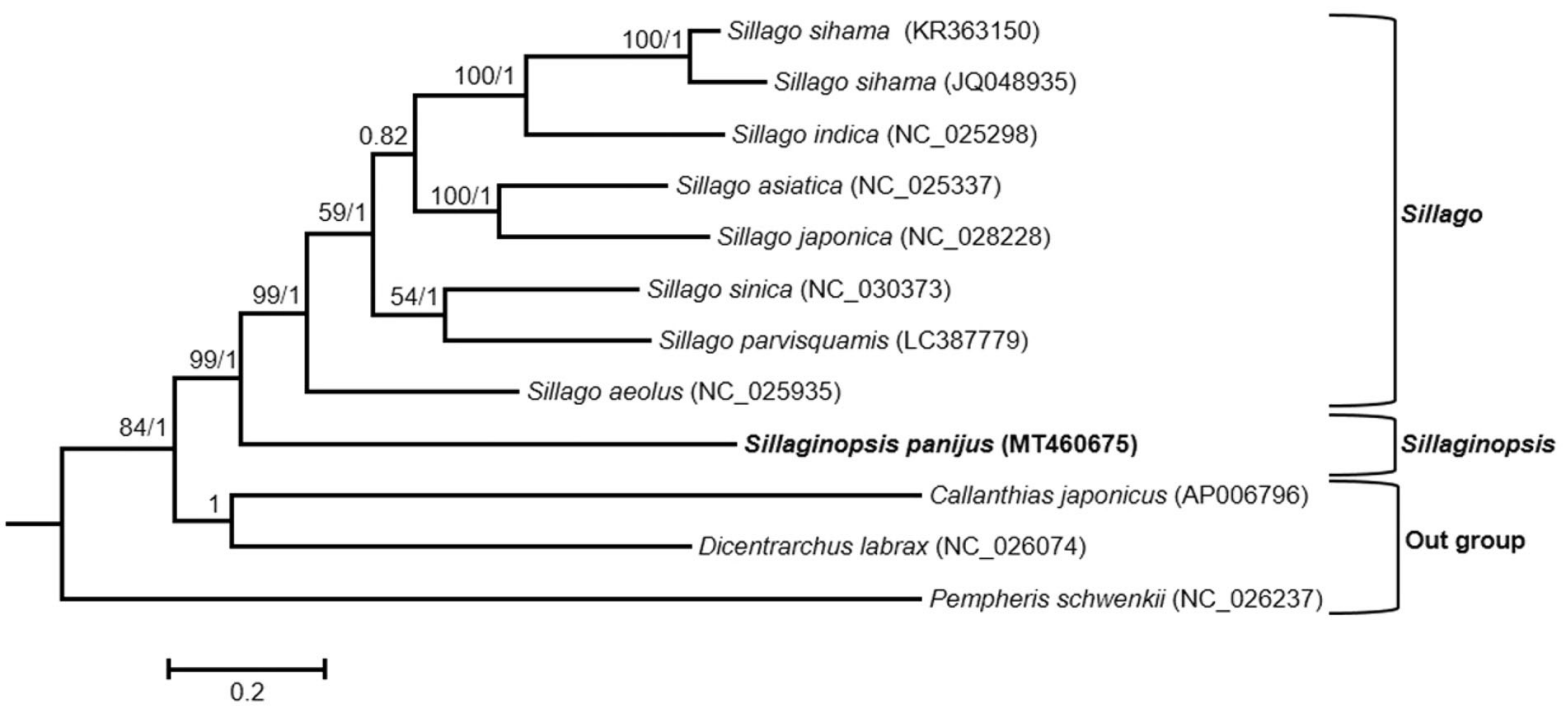
Phylogenetic tree using BI and ML methods among eight Sillaginidae species based on concatenated sequences of the 12 PCGs. The Bayesian topology was similar to the result of the maximum likelihood tree. Bootstrap support values/Bayesian posterior probabilities were displayed at branch nodes. Three species of the suborder Percoidei, *Callanthias japonicus*, *Dicentrarchus labrax* and *Pempheris schwenkii*, were selected as outgroup species.

## Data Availability

The data that support the findings of this study are publicly available in [GenBank accession number MT460675, https://www.ncbi.nlm.nih.gov/nuccore/MT460675].
